# The *Cer-cqu* gene cluster determines three key players in a β-diketone synthase polyketide pathway synthesizing aliphatics in epicuticular waxes

**DOI:** 10.1093/jxb/erw105

**Published:** 2016-03-09

**Authors:** Lizette M Schneider, Nikolai M Adamski, Caspar Elo Christensen, David B Stuart, Sonia Vautrin, Mats Hansson, Cristobal Uauy, Penny von Wettstein-Knowles

**Affiliations:** ^1^Biology Department, Copenhagen University, Copenhagen DK-2200, Denmark; ^2^Biology Department, Lund University, SW-22362 Lund, Sweden; ^3^John Innes Centre, Norwich Research Park, Norwich NR4 7UH, UK; ^4^INRA–Centre National de Ressources Génomiques Végétales, F-31326 Castanet Tolosan, France

**Keywords:** Barley, β-diketone aliphatics, carboxylesterase, *Cer-cqu* gene cluster, cytochrome P450, diketone synthase (DKS), epicuticular wax, esterified alkan-2-ols, *Hordeum vulgare*, hydroxy-β-diketones, lipase, plant apoplast, type III polyketide synthase (PKS).

## Abstract

Aliphatic compounds on plant surfaces, called epicuticular waxes, are the first line of defense against pathogens and pests, contribute to reducing water loss and determine other important phenotypes. Aliphatics can form crystals affecting light refraction, resulting in a color change and allowing identification of mutants in their synthesis or transport. The present study discloses three such *Eceriferum* (*cer*) genes in barley – *Cer-c*, *Cer-q* and *Cer-u* – known to be tightly linked and functioning in a biochemical pathway forming dominating amounts of β-diketone and hydroxy-β-diketones plus some esterified alkan-2-ols. These aliphatics are present in many Triticeae as well as dicotyledons such as *Eucalyptus* and *Dianthus*. Recently developed genomic resources and mapping populations in barley defined these genes to a small region on chromosome arm 2HS. Exploiting *Cer-c* and *-u* potential functions pinpointed five candidates, of which three were missing in apparent *cer-cqu* triple mutants. Sequencing more than 50 independent mutants for each gene confirmed their identification. *Cer-c* is a chalcone synthase-like polyketide synthase, designated diketone synthase (DKS), *Cer-q* is a lipase/carboxyl transferase and *Cer-u* is a P450 enzyme. All were highly expressed in pertinent leaf sheath tissue of wild type. A physical map revealed the order *Cer-c*, *Cer-u*, *Cer-q* with the flanking genes 101kb apart, confirming they are a gene cluster, *Cer-cqu*. Homology-based modeling suggests that many of the mutant alleles affect overall protein structure or specific active site residues. The rich diversity of identified mutations will facilitate future studies of three key enzymes involved in synthesis of plant apoplast waxes.

## Highlight

The 101kb *Cer-cqu* gene cluster, encoding CER-C (a type III chalcone synthase-like diketone synthase), CER-Q (a lipase) and CER-U (a P450 enzyme), determines significant constituents of plant apoplasts.

## Introduction

**Fig. 1.**
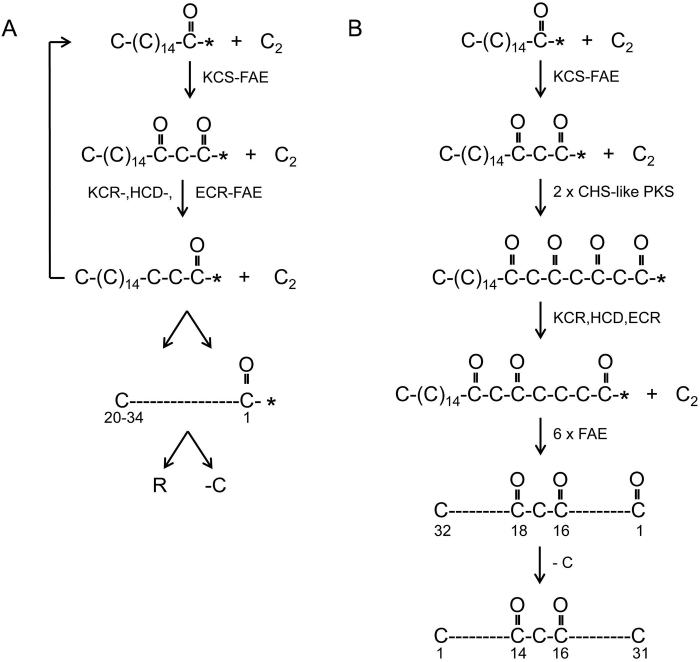
Two elongation pathways. (A). The β-ketoacyl-CoA synthase (KCS) moiety of a fatty acid elongase (FAE) complex in the endoplasmic reticulum adds C_2_-units to an acyl chain synthesized by fatty acid synthase (FAS) in a plastid. Both FAS and FAE are type II enzyme complexes as their components are coded for by distinct genes. Three subsequent reactions by the other FAE moieties [β-ketoacyl-CoA ketoreductase (KCR), β-hydroxyacyl-CoA dehydratase (HCD), β-enoyl-CoA reductase (ECR)] remove the β-oxygen. Reiteration yields C_20_-C_34_ acyl chains that serve as precursors of the reductive (R) and decarb (-C) derived FAE aliphatics. *, CoA. (B) Deduced elongation pathway giving rise to a carbon skeleton decorated with two oxygens. Addition of two C_2_-units to a β-ketoC_18_-CoA by a chalcone synthase (CHS)-like polyketide synthase (PKS) results in an acyl chain with four oxygens. That on the β-carbon is then removed by the successive action of a KCR, an HCD and an ECR. Six additional elongations of the FAE type results in a C_32_ acyl chain with oxygens on carbons 16 and 18. A decarb reaction yields the β-diketone, hentriacontan-14,16-dione.

The outermost surface of the plant’s cuticular apoplast consists of one or more of a variety of compounds. Frequently these are a mixture of very long chain aliphatics that can include various combinations of hydrocarbons, ketones, primary and secondary alcohols, aldehydes, esters and free fatty acids. They originate from 16 and 18 carbon fatty acyl chains synthesized in plastids which are transported to the endoplasmic reticulum (ER) where they can be further elongated by a fatty acyl elongase (FAE) complex consisting of a β-ketoacyl-CoA synthase (KCS) that adds a new C_2_-unit plus three additional enzymes that remove the β-oxygen to give an acyl chain which can accept another C_2_-unit. Reiteration by the FAE complex gives 20–34 acyl chains (Fig. 1A), which can then enter either a decarb pathway giving odd chain wax aliphatics or a reductive pathway yielding those with even chains that together with the remaining fatty acyl chains are transported into and/or onto the plant surface where, in some instances, crystal structures are formed (Haslam and Kunst, 2013; Yeats and Rose, 2013; Lee and Suh, 2015).

The presence of crystals on the apoplast surface contributes to the cuticle’s phenotype, which can be altered by mutations interfering with their synthesis or transport to the cuticle surface. Such mutants in barley designated *eceriferum* (*cer*) have been assigned to more than 75 complementation groups (Lundqvist and Lundqvist, 1988). The three with the most mutations are *cer-c*, *-q* and *-u* with 215, 167 and 160 alleles, respectively, representing more than one quarter of the Nordic Genetic Resource Center *cer* mutant collection. The *cer-c*, -*q* and *-u* mutations affect the wax crystals on the uppermost leaf sheaths and exposed internodes plus the spikes (glumes and lemmas), but not those on the leaf blades. While in most *cer-c* and *-q* mutants the noted cuticle surfaces are bright green (non-glaucous) in contrast to the blue (glaucous) of the wild type, those of the *cer-u* mutants have an intermediate phenotype. Included in the *cer* mutant collection are 13 apparent multiple mutants encompassing all pairwise combinations as well as seven triples. Test crosses with one of the latter (*cer-cqu.420*) revealed that any pair were within 0.0012 map units of each other implying that they were very tightly linked, forming the *Cer-cqu* gene cluster (von Wettstein-Knowles and Søgaard, 1980).

Comparisons among the waxes of the wild-type and those on the respective *cer-c*, *-q* and *-u* apoplast surfaces revealed the presence of three additional types of aliphatics to those specified above, namely β-diketones, hydroxy-β-diketones and short esterified alkan-2-ols. In addition, the mutations did not affect the wax components derived from the KCS-FAE elongation system (von Wettstein-Knowles and Søgaard, 1980). β-diketones were first identified in *Eucalyptus*, *Acacia*, *Dianthus* and *Festuca* (Horn and Lamberton, 1962), and shortly thereafter associated with long thin, crystalline tubes on the glaucous leaf surfaces (Horn *et al.*, 1964). A comprehensive survey of the β-diketones and their derivatives (hydroxyl- and oxo-β-diketones) correlated with wax ultrastructure in the Triticeae revealed their widespread distribution in this tribe (Baum *et al.*, 1980; Tulloch *et al.*, 1980). β-diketones have also been identified in *Buxus* (Dierickx, 1973), *Rhododendron* (Evans *et al.*, 1975) and *Hosta lancifolia* waxes (Jenks *et al.*, 2002). While the β-diketone aliphatics account for 50% of the wild-type spike wax load, they are absent if not dramatically reduced in the *cer-c* and *-q* mutants. In *cer-u* mutants the absence of hydroxy-β-diketones is accompanied by a compensatory increase of β-diketones (von Wettstein-Knowles, 1976). By comparison, the alkan-2-ol esters account for only ~3.7% of wild-type spike wax. They are, however, a predominant aliphatic in some *cer-c* mutants (von Wettstein-Knowles, 1971). Short esterified alkan-2-ols (C_9_–C_17_) are also found in some *Eucalyptus* waxes containing β-diketones (Horn *et al.*, 1964). As in barley, the β-diketones in three *Agropyron* species are accompanied by esterified 2-ols (primarily C_13_ and C_15_) (Tulloch, 1983). In the absence of β-diketones, short esterified alkan-2-ols occur in sorghum leaf waxes (C_9_) and in some *Papaver* fruit capsule waxes (C_11_–C_17_) (von Wettstein-Knowles *et al.*, 1984; Jetter and Riederer, 1996). With the exception of the *Papaver* waxes the above observations suggest an intimate biochemical relationship between β-diketone aliphatics and esterified alkan-2-ols.

The β-diketones noted above comprise C_29_–C_33_ homologs. Most frequently C_31_ predominates, accompanied by C_29_ and sometimes minor amounts of C_33_ homologs. Carbonyl positions can vary from 6,8 to 16,18 (von Wettstein-Knowles, 1986). In barley exploitation of *cer* mutants, radioactive precursor and inhibitor studies established that the oxygens are inserted into the carbon chains during elongation (Mikkelsen, 1979). This demonstrated that acyl chain elongation is carried out by a different system from the FAE one described above. The direction of elongation is opposite to that required for nomenclature (Fig. 1B). For example, the oxygens on carbons 14 and 16 of the predominating C_31_ homolog in barley correspond to carbons 16 and 18, respectively, during synthesis. By comparison, the associated esterified alkan-2-ols are much shorter, ranging from C_9_ to C_17_. The hydroxy groups of the esterified C_13_- and C_15_-2-ols in barley correspond to carbons 12 and 14 during synthesis, respectively (von Wettstein-Knowles, 1986).

That oxygens are present on carbons 16 and 18 during carbon skeleton formation of the β-diketones implies that the oxygen could have been on the β-carbon after the addition of a C_2_-unit to a growing acyl chain that was not removed from carbon 16 before the addition of the next C_2_-unit. This is the hallmark of polyketide synthases (PKSs; Fig. 1B). Should the same occur in the subsequent elongation then a second oxygen would be introduced on carbon 18. If followed by six elongations of the FAE type to give a C_32_ acyl chain, which is then subjected to a decarb step as in the synthesis of alkanes, the result would be the β-diketone, hentricontane-14,16-dione (Mikkelsen, 1979). PKSs are similar to FAS complexes except that they leave out one or more of the three reactions removing the β-oxygen after given elongation steps. While type I and II function as part of enzyme complexes, type III or chalcone synthase (CHS)-like PKSs are individual enzymes exhibiting substrate specificity, chain elongation and cyclization activities, all of which are attributable to the shape and size of the substrate binding pocket (Austin and Noel, 2003; Abe and Morita, 2010). That all three reactions to remove the β-oxygen are lacking in two elongation cycles of β-diketone skeleton formation suggests that a type III PKS may participate in their synthesis. Interestingly, in β-diketone synthesis only two successive elongations take place whereas most type III PKSs carry out three. Identifying the *Cer-c*, *Cer-q* and *Cer-u* genes would be a major step in determining if CER-C is indeed a CHS-like PKS and CER-U is a P450 hydroxylase as has been suggested (von Wettstein-Knowles, 2012) and would give a first indication as to the nature of CER-Q.

## Materials and methods

### Plant material and growth conditions

A set of barley cultivars Barke, Bonus, Bowman, Foma, Kristina, Maja, Morex and Quench; near-isogenic lines BW409 (carrying the *gsh6.s* allele of *Cer-c*), BW404 (*gsh1.a* allele of *Cer-q*) and BW411 (*gsh8.ag* allele of *Cer-u*); and *eceriferum* mutants (Supplementary Table S1, available at *JXB* online) were ordered from Nordic Genetic Resource Center, Alnarp, Sweden (www.nordgen.org, accessed 31 December 2015). BW409, BW404 and BW411 were each crossed to Bowman, Barke, Morex and Quench from which ten F_2_-mapping populations were produced (crosses BW404×Morex and BW411×Barke were unsuccessful). All plants and F_2_-mapping populations were planted in one liter pots and grown in a greenhouse at 18 °C under a cycle of 16-h light/8-h dark. Phenotyping was done by visual inspection of leaf sheaths at heading stage.

### DNA extraction

Leaf segments were sampled and DNA extracted using published protocols (Pallotta *et al.*, 2003) or the REDExtract-N-Amp™ Plant PCR Kit (Sigma Aldrich) using manufacturer’s instructions.

### PCR amplification and SNP marker analyses

#### Genetic mapping

PCR amplifications were performed according to the manufacturer’s protocol by using REDExtract-N-Amp PCR ReadyMix. PCR was performed at 95 °C for 3min; at (95 °C for 45s, 52–62 °C for 45s; 72 °C for 90sec) ×34 cycles; and finally at 72 °C for 5min, and gradients were run to optimize annealing temperatures (Supplementary Table S2). PCR products were analyzed on 2% agarose gels and PCR products selected for sequencing were purified using the NucleoSpin Extract II Kit (Macherey-Nagel, REF 740609). Purified PCR products were sequenced by Eurofins (www.eurofins.com, accessed 31 December 2015). Restriction endonucleases used for SNP marker analyses were identified using the NEBcutter tool (www.neb.com, accessed 31 December 2015). PCR products were digested using one unit of the appropriate restriction enzyme for up to 3h, after which samples were loaded on 2% agarose gels. SNP marker 2_1377 was analyzed with *BtsCI*, 2_0563 with *Tsp509I*, 2_0724 with *StuI*, 3_1446 with *BsaHI* and 1_0718 with *Hpy166II*.

#### Candidate gene analysis

PCR was performed using Phusion® High-Fidelity DNA Polymerase (New England Biolabs; M0530) and recommended protocol [i.e. primer concentration 500nM, 200 µM dNTPs, at 98 °C for 3min; at (98 °C for 15s, 54.5-63 °C as specified in Supplementary Table S2 and in the following for 30s; 72 °C for 90 sec] ×40 cycles; 72 °C for 5 min]. Annealing temperature was calculated using the NEB calculator (http://tmcalculator.neb.com/#!/, accessed 31 December 2015). A 2391bp fragment containing *MLOC_59804* was amplified using primer pair 59804-1-A and 59804-5-2 (63 °C); a 2215bp fragment containing *MLOC_13397* was amplified with primers 13397-01-A and 13397-3-1 (61 °C); whereas five overlapping fragments across each exon were amplified for *AK373499* (Supplementary Table S2). PCR products were visualized on 2% agarose gels, bands excised and the DNA recovered using Qiagen Gel Extraction kit (Cat. No. 28704). The BigDye® Terminator v3.1 Cycle Sequencing Kit (Cat. No. 4337456) was used to sequence the amplicons with overlapping primers (Supplementary Table S2).

### Bioinformatics analysis of the Cer-cqu region

SNP marker sequences were checked at the PGSB barley genome database (http://pgsb.helmholtz-muenchen.de/plant/barley, accessed 31 December 2015). SNP sequence positions and their corresponding Morex contigs were searched at the IPK barley BLAST server (http://webblast.ipk-gatersleben.de/barley/viroblast.php, accessed 31 December 2015). A list of all putative genes in the region between SNP marker 1_0718 and 1_1059 were found by searching the Barlex Draft Genome Explorer (http://barlex.barleysequence.org, accessed 31 December 2015). Six candidate gene models were selected based on possible gene function and were selected across the region. DNA sequences were analyzed with BioEdit (http://www.mbio.ncsu.edu/bioedit, accessed 31 December 2015), and the NCBI and Phytozome BLAST tools. Multiple polypeptide sequence alignments were performed by the Multiple Sequence Alignment program Clustal Omega (http://www.ebi.ac.uk/Tools/msa/clustalo, accessed 31 December 2015).

### BAC extraction, library preparation and sequencing

BAC HVVMRXALLrA0066C06 was identified at Centre National de Ressources Génomiques Végétales (CNRGV) Toulouse in a BAC library of barley cultivar Morex (Schulte *et al.*, 2011) via PCR using the primer pair 59804_2_F and 59804_2_R (Supplementary Table S2). Single colonies were picked and plasmid DNA extracted using Nucleobond Xtra Midi Kit from Macherey Nagel (Cat. No. 740410.100). The plasmid DNA (2 µg) was pooled with additional BAC clones and a single library was generated from this pooled DNA (40 µg) using the standard PacBio library preparation protocol (10kb libraries). This library was sequenced in one PacBio RS II SMRT Cell using the P6 polymerase in combination with C4 chemistry at the Institute for Genomic Medicine (IGM) in San Diego, USA.

### BAC assembly and annotation

The BAC assembly was performed following the HGAP workflow (https://github.com/PacificBiosciences/Bioinformatics-Training/wiki/HGAP, accessed 31 December 2015). The raw data were first cleaned from *Escherichia coli* contamination and low quality reads (read quality <0.80 and read length <500bp). The vector sequence (pSMART BAC 2.0) was cleaned after the preassembly step of the workflow. The assembly resulted in a single contig of 181kb with a mean coverage of 325× at a mean quality value of 48.54. The size of the BAC was estimated to be 180kb using gel electrophoresis, which agrees with the size of the assembly. The size of the BAC is larger than the average BAC insert size of this library (92kb), but consistent with the high variation in insert size found in this library (see Fig. 1 in Schulte *et al.*, 2011). The assembly was deposited in GenBank (accession number KU721941).

Contigs were first annotated for repetitive regions using the Triticeae Repeat Sequence Database (TREP) as well as the NCBI Nucleotide collection (nr/nt). Transposable elements were manually annotated to precisely define their features including target site duplications, long terminal repeats and short inverted terminal repeats. Regions with no hits to these repeat databases were further assessed using the Fgenesh gene prediction algorithm; the resulting predicted proteins were compared to the NCBI non-redundant protein database using blastP. Only one of the putative predicted proteins (Hv_66C06_HlyIII) had significant hits to previously annotated proteins.

### *MLOC_12151/AK373499* gene model analysis

Candidate gene *MLOC_12151* was predicted to start at the edge of a genomic contig (morex_contig_1562667), suggesting that the gene model could possibly be incomplete. A full-length cDNA, *AK373499*, with sequence similarity of 99.3% (1219/1227) to *MLOC_12151* was identified (Matsumoto *et al.*, 2011). This cDNA has an additional exon compared to *MLOC_12151* and was sequenced from barley cultivar ‘Haruna Nijo’, which explains the difference to the Morex sequence. A BLAST analysis showed that the next best hit for *AK373499* after *MLOC_12151* has a sequence similarity of 84%, suggesting that *AK373499* is allelic to *MLOC_12151*. This was further supported by the BAC sequence assembly and the RNA-seq transcriptome data, which suggests that the longer gene model (*AK373499*) is the correct one. For this reason *AK373499* instead of *MLOC_12151* was used throughout.

### RNA-Seq analysis

The publicly available dataset PRJEB12101 was obtained from the NCBI short read archive. A cDNA reference of Morex was obtained from the EnsemblPlants website (Hordeum_vulgare.082214v1.29.cdna.all.fa). As detailed above, the cDNA sequence of *MLOC_12151* was exchanged for the full-length cDNA sequence of *AK373499* in the reference and also added the sequence of the newly predicted gene *Hv_66C06_HlyIII*. The kallisto program (v-0.42.3) was used to build an index (default settings), map the sequence reads to the modified cDNA reference (default settings) and the accompanying program sleuth was used to quantify transcript abundance using 100 bootstraps (default settings).

### Analysis of the CER-C, CER-Q and CER-U proteins

#### Phylogenetic analysis:

The barley *MLOC_59804*, *MLOC_13397* and *AK373499* proteins were used as queries against the Phytozome and EnsemblPlants databases for orthologous proteins via blastp. Specifically, the Phytozome database was queried for proteins from *Brachypodium distachyon*, *Brassica rapa*, *Eucalyptus grandis*, *Medicago truncatula*, *Oryza sativa*, *Panicum virgatum*, and *Zea mays*. The EnsemblPlants database was queried for proteins from *Aegilops tauschii*, *Arabidopsis thaliana*, *Hordeum vulgare*, *Musa acuminata*, *Setaria italica*, *Sorghum bicolor*, *Triticum aestivum* and *T. urartu*. An unrelated barley protein (MLOC_66074; Photosystem I reaction center subunit III) was added to each tree to act as a root. The protein sequences were aligned using ClustalOmega (default settings) and a neighbor-joining tree was constructed using ClustalW2 (default settings; www.ebi.ac.uk/Tools/msa/clustalo, accessed 31 December 2015). Each tree was downloaded as a text file in Newick format and loaded into the Fig Tree program (v.1.4.2) for visualization.

### Modeling

Models of the predicted protein sequences were generated by comparative modeling using the ROBETTA web-server (http://robetta.bakerlab.org/, accessed 31 December 2015). Homologous structures were identified for all three proteins and used to thread the modeling algorithm. The quality of the models was assessed by manual inspection of the models and comparison to structures of homologous proteins to verify that known folds, active sites, dimer interfaces and binding pockets had been recovered realistically in the models. In addition, the distribution of the estimated errors was evaluated and verified to be located in non-core areas of the protein. The effect of the individual mutants was assessed by manual inspection of the models using PYMOL software. Mutations that substantially disturbed the integrity of the structure (e.g. by introducing clashes, hydrophilic residues in hydrophobic environments or removing stabilizing interactions) or directly interfered with the function of the protein (e.g. by substituting catalytic residues or destabilizing areas involved in binding, catalysis or homodimerisation) were annotated as having an either ‘structural’ or a more specific effect such as ‘close to binding pocket’ ‘close to catalytic site’ or ‘involved in dimer formation’. Mutations with no obvious effects on the structure or activity of the proteins were annotated as ‘Unexplained’ (e.g. a hydrophilic for hydrophilic substitution in a solvent exposed area of the protein).

## Results

### Mapping the *Cer-cqu* locus to a discrete location on chromosome arm 2HS

**Fig. 2.**
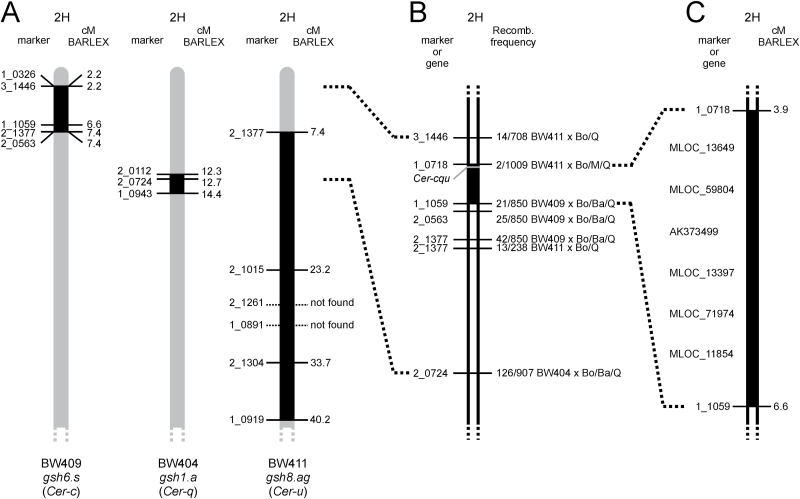
Location of *Cer-c*, *Cer-q* and *Cer-u* on barley chromosome arm 2HS. (A) Comparison of introgression regions in three different barley near-isogenic lines (Druka *et al.*, 2010). BW409 carries the *gsh6.s* allele, which is a mutation in the *Cer-c* gene (Franckowiak and Lundqvist, 2012). BW404 and BW411 contain *gsh1.a* (*Cer-q*) and *gsh8.ag* (*Cer-u*), respectively. Map positions follow the BARLEX Draft Genome Explorer (Colmsee *et al.*, 2015). (B) Mapping of the *Cer-c*, *Cer-q* and *Cer-u* loci using six SNP markers. Recombination frequency for each marker is given as number of recombinants/total progeny. The F_2_-mapping populations were made from crosses between the three near-isogenic lines and barley cultivars Bowman (Bo), Barke (Ba), Morex (M) and Quench (Q). (C) *Cer-c*, *Cer-q* and *Cer-u* were mapped between SNP markers 1_0718 and 1_1059. Six genes of unknown order were selected as *Cer-c*, *Cer-q* and *Cer-u* candidates for further analysis.

The *Cer-c*, *Cer-q* and *Cer-u* genes have previously been mapped to the short arm of chromosome 2H, 27.1 cM proximal to the RFLP marker MWG064 and 5.4 cM distal to MWG048 (Schondelmaier *et al.*, 1992). This genetic position was used together with the information of the introgression regions from three near-isogenic lines carrying mutations in *Cer-c*, *Cer-q* and *Cer-u* (Franckowiak *et al.*, 1985). Near-isogenic line BW409 carries a *Cer-c* introgression between SNP markers 1_0326 and 2_0563, BW404 carries a *Cer-q* introgression between markers 2_0112 and 1_0943, and BW411 carries a *Cer-u* introgression between markers 2_1377 and 1_0919 (Close *et al.*, 2009; Druka *et al.*, 2010). The three introgressed regions are partly overlapping (Fig. 2A). Six SNP markers polymorphic across the introgressed regions (Fig. 2B) were used in the analyses of ten F_2_-mapping populations, which were created from crosses of the three near-isogenic lines to barley cultivars Bowman, Barke, Morex and Quench.

That the three genes are located extremely close to each other (von Wettstein-Knowles and Søgaard, 1980) infers that the different mapping populations could be pooled to identify a unique position for them on chromosome arm 2HS. More than 3000 F_2_ plants were grown, phenotyped and subsequently analyzed with the six SNP markers. The results from the SNP marker analyses of the F_2_-mapping populations defined the location of *Cer-c*, *Cer-q* and *Cer-u* to an interval flanked by distal marker 1_0718 and proximal marker 1_1059 (Fig. 2C).

These two markers are located on morex_contig_6591 and morex_contig_276408, respectively, corresponding to a region of 2.68 cM based on the barley POPSEQ map (Mascher *et al.*, 2013). The region contains 582 gene models according to EnsemblPlants (Bolser *et al.*, 2015) while 194 gene models are predicted in the BARLEX Draft Genome Explorer (Colmsee *et al.*, 2015), 72 of which have been annotated. Based on these putative functions and the previous predictions from von Wettstein-Knowles (2012), five genes were selected as likely candidates for *Cer-c* and *Cer-u*; *MLOC_11854* predicted to encode a transferase, *MLOC_59804* which contains a type III polyketide-synthase domain, and *MLOC_13649, MLOC_12151*/*AK373499* (see methods) and *MLOC_71974* which belong to the cytochrome P450 family.

**Fig. 3.**
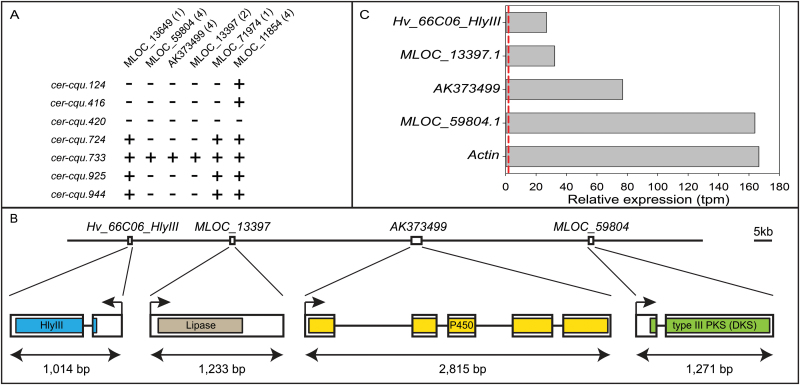
*Cer-cqu* candidate genes, barley BAC sequence and gene expression. (A) PCR amplification of six candidate genes using DNAs from barley *cer-cqu* triple mutants as templates. The inability to amplify *MLOC_59804*, *MLOC_13397* and *AK373499* from six mutants pinpointed these as *Cer-c*, *Cer-q* and *Cer-u* candidates. The number of primer pairs used with each gene is in parenthesis. +, gene amplified; -, gene not amplified. (B) Proposed order and gene models of the four genes identified on barley BAC HVVMRXALLrA0066C06. Regions encoding conserved domains are colored. Bar, 5kb. (C) Relative expression of the four genes based on RNA-seq analysis of barley flag leaf sheath (SRA PRJEB12101). *Actin* (*MLOC_54382.1*) is included as a reference gene. The dashed red line corresponds to a transcripts per million (tpm) value of 2, commonly used as the cut-off for real expression over noise.

To test the five candidate genes for the likelihood of them being *Cer-c* or *Cer-u*, the seven apparent triple *cer-cqu* mutants were exploited. Six of them had been induced by fast neutrons and one (*cer-cqu.124*) by gamma rays suggesting their potential to be deletions (von Wettstein-Knowles and Søgaard, 1980). Two genes (*MLOC_59804* and *AK373499*) could not be amplified in six of the seven triple mutants. An additional gene, encoding a lipase (*MLOC_13397*), was selected as a putative candidate for *Cer-q* based on its proximity to the *Cer-c* and *Cer-u* candidates in the POPSEQ map. This gene also failed to amplify in the same six triple mutants as *MLOC_59804* and *AK373499*. It is worth noting that the three genes were amplified in the triple mutant *cer-cqu.733*. This inconsistency is most likely explained by a low degree of seed mix-up which inevitably occurs in historic mutant collections over the years (Zakhrabekova *et al.*, 2012; Dockter *et al.*, 2014). Taken together, the deletion analysis of the *cer-cqu* mutants, along with their predicted functions, pinpointed these three genes as candidates for *Cer-c*, *Cer-q* and *Cer-u* (Fig. 3A).

### BAC and candidate gene annotation

To determine the proximity of the three candidate genes within the barley physical sequence, we screened a barley bacterial artificial chromosome (BAC) library of cultivar Morex via PCR (see ‘Materials and methods’). We identified BAC clone HVVMRXALLrA0066C06 and successfully amplified all three candidate genes from the BAC template. Using PacBio reads we performed a sequence assembly of BAC HVVMRXALLrA0066C06, thus obtaining a single contig of 181,647 base pairs in size (GenBank accession KU721941). We first annotated the repetitive portion of the BAC using publicly available databases (TREP, NCBI). Approximately 67% of the BAC sequence (122067bp) consisted of transposable elements (TEs), which is lower than the estimated average of ~85%, suggesting a gene-rich region. Among the TEs present on the BAC are three complete long terminal repeat (LTR) retrotransposons belonging to the Ty1-*copia* family. These elements are over 97% identical to each other, suggesting very recent local duplications.

We used gene prediction algorithms to identify open reading frames on the BAC (Solovyev *et al.*, 2006). In addition to the three candidate genes *MLOC_59804*, *MLOC_13397* and *AK373499*, we identified a fourth gene that encodes a member of the haemolysin III family predicted to function as an integral membrane channel protein. This fourth gene, named *Hv_66C06_HlyIII* hereafter, appears to be novel as no matching barley gene model or full-length cDNAs could be detected in public databases. No other genes were predicted on the BAC.

The order of the four genes was determined to be *Hv_66C06_HlyIII*, *MLOC_13397*, *AK373499* and *MLOC_59804*, with 27.3, 49.2 and 46.5kb, respectively, between the termination codon of the preceding and the start codon of the following gene (Fig. 3B). By disregarding TEs, the physical interval between the four genes was 13.7, 12.7 and 13.7kb, respectively. The accumulated distance between the *Cer-cqu* genes was 101kb and 31.7kb without TEs.

We surveyed the expression of the genes identified on the BAC using publicly available transcriptome data (PRJEB12101) of leaf sheath tissue from barley cultivar Foma collected at growth stage 43 (flag leaf sheath just visibly swollen). At this developmental stage barley has visible wax deposition on the leaf sheath. We used the kallisto-sleuth package (Bray *et al.*, 2015) to map reads and quantify transcript abundance. Analysis of the RNA-seq data confirmed the gene models for *MLOC_59804*, *MLOC_13397* and *Hv_66C06_HlyIII* as well as the full-length cDNA model *AK373499* including exon 1, which is missing from the Morex whole genome sequence assembly (*MLOC_12151*; see ‘Materials and methods’) (Fig. 3B). All three candidate genes as well as *Hv_66C06_HlyIII* are highly expressed in flag leaf sheaths, with *MLOC_59804* being almost as highly expressed as *Actin* (*MLOC_54382*; Fig. 3C). These results agree with the expected expression patterns of candidate genes for *Cer-c*, *-q* and *-u*.

### Independent validation of *Cer-c*, *-q* and *-u* candidate genes

To validate the three candidate genes we examined 57, 57 and 54 additional *cer-c*, *-q* and *-u* mutants, respectively. Each candidate gene, including >400bp upstream of the start codon and >100bp downstream of the termination codon, were amplified and sequenced. Mutations were identified in 53, 52 and 54 *cer-c*, *-q* and *-u* mutants for the candidate genes *MLOC_59804*, *MLOC_13397* and *AK373499*, respectively (Tables 1–3). In the majority of cases, these mutations led to a non-synonymous amino acid change (46 *cer-c*, 37 *cer-q* and 32 *cer-u*), while only a small number resulted in premature termination codons (two, five and nine) or caused a frameshift in the protein (three, six and four). Mutations in canonical splice sites were only observed in four *cer-u* mutants, consistent with the fact that *MLOC_59804* (*Cer-c*) has only two exons and that *MLOC_13397* (*Cer-q*) lacks an intron altogether. No amplicons could be obtained for the corresponding gene in two *cer-c*, four *cer-q*, and two *cer-u* mutants, although the other genes could be amplified, suggesting that these are single gene deletions.

**Table 1.** Mutations in barley MLOC_59804 (Cer-c) organized on the basis of their position in the gene

*cer*	Mutagen^a^	Year	C^b^	CDS position	Protein	Effect on enzyme
1131^c^	EMS	1969	K	G(−18)A	5’ UTR	---
36	γ-rays	1956	B	1bp del (78)	frame (26>)	---
511	PMS	1963	F	C121T	P41S	Near active site pocket
902	iPMS	1970	B	A128G	Y43C	Near substrate binding tunnel
1334	NaN3	1975	B	C143T	T48I	Near substrate binding tunnel
945	EMS	1969	B	A145T	K49*	---
987	ethylene oxide	1971	B	T178A	F60I	Near substrate binding tunnel
544	NMUN	1965	F	G191A	C64Y	Near active site pocket
890	ethylene oxide	1970	B	C214T	R72C	Near substrate binding tunnel
223	ethylene imine	1959	F	T221C	F74S	Near substrate binding tunnel
171	PDADE	1965	B	T305A	V102E	Near substrate binding tunnel
999	iPMS	1977	B	A310T	K104*	---
469	EMS + neutrons	1963	F	C404T	T135I	Near active site pocket
760	ethylene oxide	1968	B	C431A	A144D	Structural
405	nBMS	1962	F	C478T	R160C	Dimerization
379	nBMS	1962	F	T494A	L165H	Near active site pocket
1329	EMS	1975	B	G508A; A986G	A170T; K329R	Near active site pocket
1348	NaN3	1976	B	C518T	S173F	Near active site pocket
73	ethylene imine	1958	B	5bp del (522–526)	frame (175>)	---
95	γ-rays	1961	B	5bp del (522–526)	frame (175>)	---
361	EMS	1962	F	T590A	L197H	Near substrate binding tunnel
1386	EMS	1975	B	G611A	S204N	Near substrate binding tunnel
915	iPMS	1970	B	G664A	D222N	Near substrate binding tunnel
881	EMS	1968	B	G668A	G223E	Near substrate binding tunnel
502	ethylene imine	1964	F	A682T	I228F	Structural
471	EMS + neutrons	1963	F	A682T	I228F	Structural
44	X-rays	1957	B	A713T	E238V	Structural
206	X-rays	1959	F	A713T	E238V	Structural
703	Neutrons	1967	B	A746G	Q249R	Dimerization
7	Spontaneous	1950	B	A747T	Q249H	Dimerization
3	X-rays	1943	B	A747T	Q249H	Dimerization
335^3^	EMS	1962	F	A747T	Q249H	Dimerization
1359	EMS	1976	B	C775T	L259F	Near active site pocket
61	ethylene imine	1958	B	T776A	L259H	Near active site pocket
673	ethylene imine	1966	B	T836A	L279Q	Near active site pocket
89	γ-rays	1958	B	T839A	V280E	Near active site pocket
1448	NaN3	1977	B	G841A	A281T	Near substrate binding tunnel
1757	NaN3	1980	B	G841A	A281T	Near substrate binding tunnel
748	ethylene oxide	1967	B	T916A	W306R	Structural
1703	NaN3	1979	B	C929T	P310L	Near active site pocket
499	EMS	1964	F	G941A	G314D	Near substrate binding tunnel
596	ENU	1967	F	A943T	I315F	Near active site pocket
270	EMS	1962	F	T947G	M316R	Near active site pocket
332	γ-rays + EMS	1961	F	T947G	M316R	Near active site pocket
238	alpha-epichlorohydrine	1959	F	T1010A	V337E	Near active site pocket
240	Glycidol	1959	F	T1010A	V337E	Near active site pocket
153^3^	ethylene oxide	1970	B	T1010A	V337E	Near active site pocket
635	ethylene imine	1970	B	A1016T	N339I	Structural
698	ethylene imine	1966	B	T1020A	F340L	Structural
413	γ-rays	1963	F	C1026A; A1027C	N342K	Active site (Catalytic residue)
284	EMS	1960	F	T1102A	W368R	Dimerization
520	EMS	1965	F	C1136T	T379I	Dimerization
455	EMS	1963	F	C1153T	L385F	Structural
609	EMS	1963	F	C1153T	L385F	Structural
115	γ-rays	1957	E	deletion	---	---
870	Neutrons	1969	B	deletion	---	---

^a^ EMS, ethyl methanesulfonate; PMS, n-propyl methanesulfonate; iPMS, isopropyl methanesulfonate; hPMS, hydroxy-propyl methanesulfonate; NaN3, sodium azide; MNU, N-methyl-N-nitrosourea; nBMS, n-butyl methanesulfonate; ENU, N-ethyl-N-nitrosourea; ENUR, N-ethyl-N-nitrosourethane; NMUN, N-methyl-N-nitrosourethane; PDADE, propane disulfonic acid diethyl ester.

^b^ Cultivar (B, Bonus; E, *erectoides-a.23*; F, Foma, K, Kristina).

^c^ Accessions originally classified as *cer-q* mutants.

**Table 2.** Mutations in barley MLOC_13397 (Cer-q) organized on the basis of their position in the gene

*cer*	Mutagen^a^	Year	C^b^	CDS position	Protein	Effect on enzyme^c^
1238	iPMS	1972	K	T2A	M1K	---
295	EMS	1960	F	C38T	A13V	*Unexplained*
128	γ-rays	1964	B	1bp del (42)	frame (14>)	---
1320	X-rays	1975	B	A65G	Y22C	*Unexplained, but Cys*
42	X-rays	1957	B	1bp del (68)	frame (23>)	---
1375	NaN3	1976	B	G92A	G31D	*Unexplained (surface*)
1439^d^	EMS	1976	B	G92A	G31D	*Unexplained (surface*)
1143	EHMS	1969	K	A101G	E34G	*Unexplained (surface*)
82	X-rays	1948	B	1bp del (202)	frame (68>)	---
900	iPMS	1970	B	A269T	H90L	Active site pocket + Structural
754	ethylene oxide	1968	B	T280A	F94I	Active site pocket + Structural
683	ethylene imine	1966	B	T283A	C95S	Active site pocket
566	ENU	1966	F	A290T	E97V	Active site pocket
1225	EMS	1972	K	G293A	S98N	Active site pocket + Structural
1243	iPMS	1972	K	G317A	R106H	Active site pocket + Structural
103	X-rays	1954	E	1bp del (339)	frame (114>)	---
1283	EMS	1974	K	G385A	E129K	Unexplained (surface)
1412	iPMS	1977	B	A386G	E129G	Unexplained (surface)
395	EMS + neutrons	1962	F	G435A	W145*	---
401	Neutrons	1967	F	G435A	W145*	---
262	ethylene imine	1960	F	T437A	V146E	Structural
218	ethylene imine	1959	F	1bp del (442)	frame (148>)	---
483	PMS	1963	F	T461A	L154H	Structural
488	EMS + neutrons	1963	F	T461A	L154H	Structural
1193	iPMS	1971	K	A466T	K156*	---
1748	NaN3	1980	B	G502A	D168N	Active site pocket (next to catalytic residue)
310	EMS	1960	F	G506A	S169N	Active site pocket (catalytic residue)
246	ethylene imine	1959	F	C509T	A170V	Active site pocket (next to catalytic residue)
239	Glycidol	1959	F	G514T	G172C	Active site pocket
1141	hPMS	1969	K	G515A	G172D	Active site pocket
1167	MNU	1970	K	G515A	G172D	Active site pocket
341	γ-rays + EMS	1962	F	C600A	F200L	Active site pocket
1459	NaN3	1978	B	G607T	G203W	Structural
176	PDADE	1965	B	T655C	F219L	Active site pocket
1368	iPMS	1976	B	T667A	W223R	Active site (substrate binding pocket)
1358	EMS	1976	B	G669A	W223*	---
1742	EMS	1979	B	G670A	V224M	Structural
320	ethylene imine	1961	F	A677T	K226M	Structural (surface)
636	ethylene imine	1970	B	T688A	F230I	Structural
1345	NaN3	1975	B	C733T	P245S	Structural
1400	EMS	1977	B	C733T	P245S	Structural
555	γ-rays	1966	F	C811T	R271W	Structural
1499	NaN3	1979	B	G814A	G272R	Active site pocket
574	PDADE	1966	F	T897A	H299Q	Active site pocket (catalytic residue)
1281	X-rays	1974	K	3bp del (923–925)	R308-;A309P	*Structural (surface*)
1128	EMS	1969	K	C1040T	P347L	*Structural*
1706	NaN3	1979	B	G1057A	D353N	*Unexplained*
400	EMS	1963	F	A1111T	K371*	---
1209	X-rays	1972	K	2bp del (1138–1139)	frame (380>)	---
425	γ-rays	1963	F	deletion	-	---
536	Neutrons	1965	F	deletion	-	---
548	MNU	1965	F	deletion	-	---
876	Neutrons	1969	B	deletion	-	---

^a^ EMS, ethyl methanesulfonate; PMS, n-propyl methanesulfonate; iPMS, isopropyl methanesulfonate; hPMS, hydroxy-propyl methanesulfonate; NaN3, sodium azide; MNU, N-methyl-N-nitrosourea; ENU, N-ethyl-N-nitrosourea; EHMS, ethylhydroxy-ethanesulfonate; PDADE, propane disulfonic acid diethyl ester; mEMS, 2-methoxyethyl methane-sulfonate.

^b^ Cultivar (B, Bonus; E, *erectoides-a.23*; F, Foma, K, Kristina).

^c^ Substitutions in low confidence regions of model are shown in italics.

^d^ Accessions originally classified as *cer-c* mutants.

**Table 3.** Mutations in barley AK373499 (Cer-u) organized on basis of their position in the gene

*cer*	Mutagen^a^	Year	C^b^	CDS position	Protein	Effect on enzyme
1137	ethylene imine	1969	K	A58T	R20*	---
371	ethyl ethanesulfonate	1962	F	T74A	L25Q	Structural
1282	EMS	1974	K	G152A	G51E	Structural
1825	iPMS	1980	B	G167A	C56Y	Substrate binding
1108	MNU	1968	K	C178T	L60F	Substrate binding
689	ethylene imine	1966	B	G191A	R64Q	Unexplained
895	iPMS	1970	B	A218T	H73L	Structural
443	nBMS	1963	F	C241T	P81S	Substrate binding
986	ethylen oxide	1971	B	T256A	W86R	Structural
1496	NaN3	1979	B	G258A	W86*	---
525	EMS	1965	F	G258A	W86*	---
1146	EHES	1969	K	G272A	G91E	Structural
542	MNU	1965	F	T298A	W100R	Structural
505	PDA	1964	F	T335A	V112E	Structural
1309	iPMS	1974	K	T395A	L132*	---
1202	EMS	1971	K	G397A	E133K	Structural
338	EMS	1961	F	T464A	I155N	Heme binding
901	iPMS	1970	B	C473G; T475A	A158G;F159I	Heme binding
304	PMS	1960	F	2^nd^ intron GT	splice donor	---
1165	ENU	1970	K	10^th^ bp 2^nd^ intron	Intron	---
307	Myleran	1960	F	G538T	E180*	---
107	X-rays	1955	E	1bp del (607)	frame (203>)	---
556	γ-rays	1966	F	1bp del (647)	frame (216>)	---
376	nBMS	1962	F	C707T	A236V	Substrate binding
825	EMS	1969	B	C707T	A236V	Substrate binding
237	ECH	1959	F	T718A	F240I	Substrate binding
1114	γ-rays	1969	K	T719C	F240S	Substrate binding
387	EMS+	1962	F	G724A	D242N	Substrate binding
855	EMS	1969	B	3^rd^ intron AG	splice acceptor	---
281	EMS	1960	F	G747A	R249R	No effect (synonymous)
452	EMS	1963	F	C776T	T259I	Structural
1402	EMS	1977	B	C776T	T259I	Structural
69	ethylene imine	1958	B	G780A	W260*	---
1177	γ-rays	1970	K	2bp del (783–784)	frame (261>)	---
613	EMS	1967	F	A805T	K269*	---
1759	NaN3	1980	B	G814T	E272*	---
1215	Neutrons	1972	K	2bp del (940-A941)	frame (314>)	---
606	ethylene imine	1966	F	G970A	E324K	Substrate binding
677	ethylene imine	1966	B	T1010C	L337P	Structural
1370	NaN3	1976	B	4^th^ intron GT	splice donor	---
21	γ-rays	1955	B	3^rd^ bp 4^th^ intron	Intron	---
1340	NaN3	1975	B	4^th^ intron AG	splice acceptor	---
570	ENUR	1966	F	A1141G	R381G	Structural
491	ethylene imine	1964	F	A1171T	R391W	Structural
776	EMS	1968	B	C1237T	P413S	Substrate binding
789	ethylene imine	1968	B	A1253C	H418P	Structural
457	Neutrons	1963	F	A1253C	H418P	Structural
1327	EMS	1975	B	C1309T	Q437*	---
177	PDA	1965	B	T1352A	L451Q	Heme binding
887	ethylene imine	1970	B	T1357A	F453I	Heme binding
1237	iPMS	1972	K	T1358A	F453Y	Heme binding
737	ethylene oxide	1968	B	T1433C	L478P	Structural
58	ethylene oxide	1958	B	Deletion	-	---
468	PMS	1963	F	Deletion	-	---

^a^ EMS, ethyl methanesulfonate; PMS, n-propyl methanesulfonate; iPMS, isopropyl methanesulfonate; NaN3, sodium azide; MNU, N-methyl-N-nitrosourea; nBMS, n-butyl methanesulfonate; ENU, N-ethyl-N-nitrosourea; ENUR, N-ethyl-N-nitrosourethane; EHMS, ethylhydroxy-ethanesulfonate; PDS, propane disulfonic acid; ECH, alpha-epichlorohydrine.

^b^ Cultivar (B, Bonus; E, *erectoides-a.23*; F, Foma; K, Kristina).

No mutations could be detected in *MLOC_59804* and *MLOC_13397* in four *cer-c* and five *cer-q* mutants, respectively. For these nine mutants the other two candidate genes (e.g. *MLOC_13397* and *AK373499* for a *cer-c* mutant) were amplified and sequenced. For three *cer-q* mutants a mutation could be detected in *MLOC_59804*, while for one *cer-c* mutant a mutation in *MLOC_13397* was identified. No mutation in any of the three genes was detected for the remaining five mutants. This probably reflects the challenge of keeping phenotypically similar mutant accessions separate over decades. Similarly, in a few cases the mutations identified were identical between two mutant lines (eight, five and four cases for *MLOC_59804*, *MLOC_13397* and *AK373499*, respectively). These mutations occur in lines which differ in the cultivar, the isolation year, the mutagen used or a combination of these, suggesting that they are independent mutation events.

**Fig. 4.**
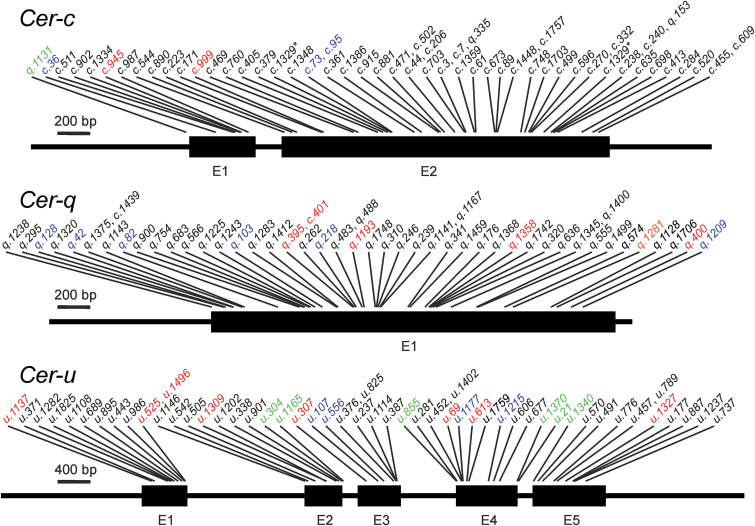
Positions of identified mutations in the barley *Cer-c*, *Cer-q* and *Cer-u* genes. Most were point mutations leading to non-synonymous substitutions of amino acids (mutation names in black). Others included nonsense mutations (red), small deletions of 1–3bp (blue), or point mutations located upstream of the ATG start codon or in intron splice sites (green). Mutation *cer-c.1329* (marked with an asterisk) contained two mutations in different parts of *Cer-c*. Further details are in Tables 1–3 and Supplementary Table S1. E, exon.

Taken together, the identification of over 50 independent mutants across each candidate gene provides strong evidence that *MLOC_59804*, *MLOC_13397* and *AK373499* encode CER-C, -Q and -U, respectively (Table 4, Fig. 4).

**Table 4.** Summary of mutation types identified in cer mutants

Mutant	Candidate gene	Mutants studied	Mutations in candidate	Non- synonymous	PTC^a^	Frameshift	Splice	Synonymous/ intron/ UTR	Whole gene deletion	No mutations detected	Misclassified^b^	Shared between lines
*Cer-c*	*MLOC_59804*	59	54	48	2	3	0	1	2	3	3	8
*Cer-q*	*MLOC_13397*	55	49	38	5	6	0	0	4	2	1	5
*Cer-u*	*AK373499*	54	52	32	9	4	4	3	2	0	0	4

^a^ PTC, premature termination codon.

^b^ Lines originally classified as mutants for alternative locus (*cer-q* or *cer-c*).

### Phylogenetic analysis of the CER-C, -Q and -U proteins

To assess the phylogeny of the CER-C, -Q and -U proteins we performed BLASTP analyses using publicly available protein sequences. Proteins from mono- and dicotyledonous species (including *Eucalyptus*) were aligned and phylogenetic trees constructed for each of the three genes. As expected the most closely related orthologs among the grasses belonged to members of the Triticeae tribe (*Triticum aestivum*, *Aegilops tauschii*; Supplementary Figs S1–S3). Closely related orthologs were also identified in foxtail millet (*Setaria italica*), switchgrass (*Panicum virgatum*), *Brachypodium* and sorghum, although generally these species formed a parallel clade relative to the Triticeae CER-C, -Q and -U proteins. The closest rice and *Brachypodium* orthologs occur in more distantly related clades and are more similar to other barley proteins than to CER-C, -Q and -U (Supplementary Figs S1–S3). This was confirmed through a reciprocal BLAST analysis for the closest *Brachypodium* and rice orthologs to barley.

### Comparative modeling for CER-C, CER-Q and CER-U

To assess the biophysical effects of the mutations we prepared models of the CER-C, -Q and -U proteins and mapped the identified mutations onto these. The models were prepared using the comparative modeling algorithms implemented at the Robetta web-server (Song *et al.*, 2013). For all three proteins, clades of existing structures with homology to the proteins were identified and served as templates in the subsequent modeling.

**Fig. 5.**
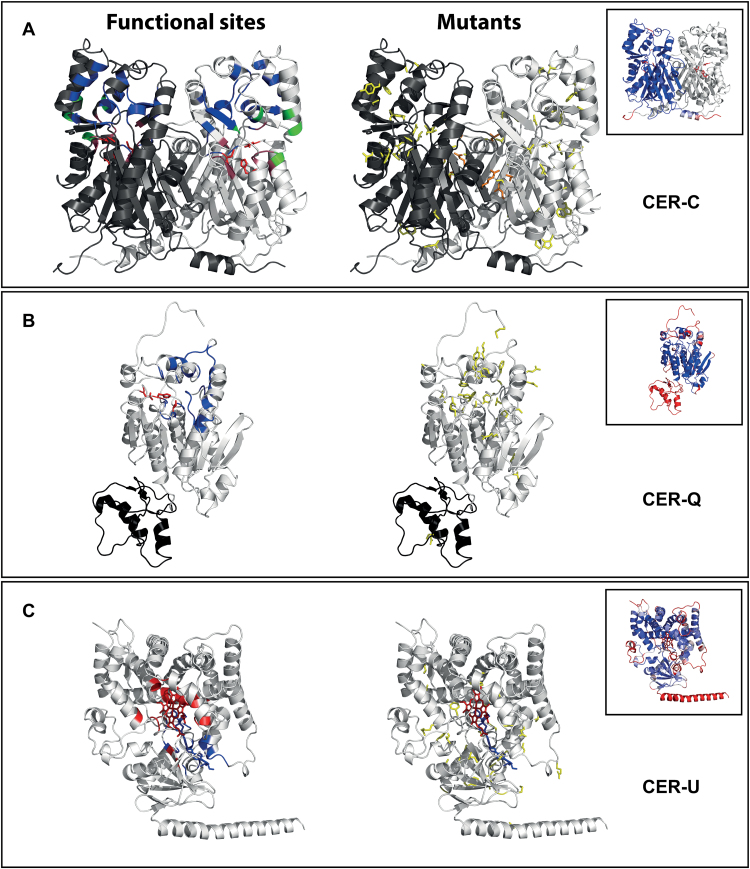
3D models of CER-C, CER-Q and CER-U. The proteins are represented in backbone cartoon with backbone colors representing functional sites in the cartoon models to the left and amino-acid residues affected by the mutations represented as yellow sticks in the models to the right. (A) CER-C represented in proposed homodimer form with subunits in black and white. To the left, the catalytic triad is represented in red sticks while the backbone of the residues forming the pockets associated with activity are colored purple (active site pocket) except for the catalytic residues in red; blue (substrate binding tunnel) and green (CoA-binding tunnel). (B) CER-Q with the lipase domain 1 in white and domain 2 in black. To the left, the active site residues are represented as red sticks and the backbone of the residues delineating the active site pocket are colored blue. (C) CER-U with heme included in the heme-binding pocket in red sticks. To the left, the backbone of the residues involved in heme binding are colored red while the substrate binding tunnel is colored blue. In the inserts the models are colored according to the predicted model error from blue (low error) to red (high error). PDB models and Pymol session files showing mutant names are accessible in the models (Supplementary Data S1–S3) and Pymol sessions (Supplementary Data S4–S6).

CER-C was modeled over the thiolase fold containing *Marchantia polymorpha* stilbene carboxylate synthase 2 (confidence score: 0.93; sequence identity: 39%; PDB code: 2p0u; Fig. 5A) reproducing a homodimeric thiolase-fold structure as expected for a CHS-like synthase (Austin and Noel, 2003). The model faithfully reproduced the position of the catalytic triad (Cys168, His309 and Asn342; Fig. 5A, red sticks) in a hydrophobic active site pocket (Fig. 5A, red backbone) connected to the exterior through a CoA-binding tunnel (Fig. 5A, green backbone) and a long substrate binding tunnel (blue backbone) traversing the protein to the surface opposite the entry of the CoA binding pocket. Out of the 48 mutants encoding non-synonymous substitutions, one targeted the catalytic Asn342, 30 affected other aspects of the active site or binding pockets, ten were predicted to affect the overall stability of the protein (Table 1, ‘structural’) and seven destabilized the interface between the homodimers (Table 1, ‘dimerization’). Only four mutants were predicted to affect surface residues, and they all either introduced cysteines prone to forming unfavorable disulfides, localized near the binding tunnels or featured destabilizing hydrophilic for hydrophobic substitutions (Y43C, L279Q, N339I and F340L). Thus, the CER-C model supports the conclusion that the *cer-c* mutants are caused by mutations in the CHS-like synthase encoded by *MLOC_59804*.

Modeling of CER-Q identified two distinct domains produced with two different clades of template structures (sequence coverage domain1/domain2: 1–328/329–410; confidence scores: 0.83/0.33; sequence identity: 25%/17%; PDB codes: 1lzl/2vsa; Fig. 5B). Domain 1 constituted an α/β hydrolase fold with the bacterial heroine esterase as closest structural homolog (Zhu *et al.*, 2003), while the closest structural homologs of domain 2 were lectin-containing ricin-like toxin domains (Treiber *et al.*, 2008). Despite the lower overall quality of the model, the α/β hydrolase core of domain 1 was modeled with high confidence and a conserved catalytic triad located at the bottom of a substrate binding pocket (Ser169, Asp266, His299; Fig. 5B, red sticks) (Holmquist, 2000). The low confidence of domain 2 was mostly due to the inability to confidently model the relative orientation of the two domains and a poor modeling of the cap-domain on top of the active site pocket (Fig. 5B, inset). The mutations identified in the *cer-q* mutants included the two catalytic residues Ser169 and H299, 13 residues in or near the active site pocket and eight substitutions resulting in a general destabilization of the protein (Table 2; Supplementary Table S1). The model failed to provide an explanation for eight of the 37 non-synonymous substitutions identified in *MLOC_13397*, however these predominantly occurred in domain 2 and other poorly predicted parts of the model (Table 2, Supplementary Table S1). Thus, analysis of the mutants across the MLOC_13397 model supports that *Cer-q* encodes a lipase.

The closest structural homologs of CER-U were all identified among the cytochrome P450 family of proteins (confidence score: 0.68; sequence identity: 17%; PDB code: 4k0f; Fig. 5C). The CER-U model included a heme pocket complete with the conserved Cys460 thiol oriented towards the point where the heme iron would be positioned. The heme-binding pocket was connected to the solvent via a substrate binding tunnel similar to other known P450 proteins. Interestingly, residues 5–26 of the C-terminal helix was predicted to constitute a trans-membrane helix using the TMHMM prediction algorithm (Krogh *et al.*, 2001). Out of the 32 non-synonymous substitutions in CER-U (Table 3), 15 were predicted to disturb the area around the heme binding pocket or the substrate binding pocket, while 16 were predicted to destabilize the protein in general. The only mutation without an obvious impact on the activity of CER-U, *cer-u.689*, was located in an area of low model quality (Table 3). Thus, this analysis supports the conclusion that *AK373499* (*Cer-u*) encodes a cytochrome P450. The models in pdb file format along with Pymol session files, showing the models with mutant residues labeled with amino acid residue substitution and source mutant strain name, are accessible given in Supplementary Data S1–S6.

## Discussion

### The *Cer-cqu* gene cluster

*Cer-cqu* was the first potential gene cluster or multifunctional gene found in plants (von Wettstein-Knowles and Søgaard, 1980). These designations were based on the close linkage, two orders of magnitude smaller than for any other barley genes at the time, and their action in a distinct secondary metabolic pathway. With the present identification of three adjacent, non-homologous genes within 101kb, the gene cluster designation has now been confirmed. Many other clusters have been found in recent years, and all have in common that they affect secondary metabolic pathways involved in biotic or abiotic defense (Boycheva *et al.*, 2014), as is true for the *Cer-cqu* cluster. According to Nützmann and Osbourn (2014), one gene in such a cluster is the signature enzyme defining the unique metabolic structure. For the *Cer-cqu* cluster this is *Cer-c* encoding a type III chalcone synthase-like protein. Signature genes in plants seem to have evolved from genes participating in primary metabolism via gene duplication and subsequent neofunctionalization. The progenitor of CER-C is presumably the β-ketoacyl-acyl carrier protein synthase KAS III of FAS (Austin and Noel, 2003). These signature genes recruit additional genes, in this case *Cer-q* and *Cer-u*, a lipase and P450, respectively, through an unknown mechanism, thus creating a gene cluster.

Is the cluster likely to include additional genes? On the *Cer-c* end of the cluster the BAC extends for more than 30kb, all of which has been annotated as repetitive sequence. At the other end of the BAC, *Hv_66C06_HlyIII* is 21kb from the BAC end and only 27kb away from *Cer-q*. The latter very small distance infers that quadruple deletion mutants should have been found if *Hv_66C06_HlyIII* were part of the *Cer-cqu* cluster. Such mutants were not identified during the allele testing of the 872 *cer* mutants affecting wax on the leaf sheaths, internodes and spikes (Lundqvist and Lundqvist, 1988). Combined, the above evidence supports the notion that additional genes are not present in the *Cer-cqu* cluster, or if they are, they are not contiguous.

To distinguish the *Cer-cqu* gene cluster pathway from those determined by other type III PKSs, CER-C has been designated β-diketone synthase (DKS). More than 900 type III PKSs representing 20 different functionalities, had been identified by 2010 (Abe and Morita, 2010). DKS has only two of the three attributes of a typical type III PKS enzyme, namely substrate specificity and elongation activity carrying out two extensions. Three condensations are the most common, with a range of 1–8 (Abe and Morita, 2010). The plant enzymes benzalacetone synthase and curcuminoid synthase do not carry out cyclization reactions (Abe and Morita, 2010). Given the lack of cyclic *in vivo* wax products, the same may be true for DKS.

### The DKS polyketide pathway for wax aliphatics

A major question to be answered about the β-diketone pathway is whether the additional six elongations giving a C_32_ chain (Fig. 1) are also carried out by the DKS or rather by an FAE type KCS. In this connection it is interesting to note that of the 50 *Cer* genes that visually affect spike waxes and hence the β-diketone aliphatics, mutants of the 29 studied do not modify the β-diketone chain length distributions, revealing that elongation is not influenced. Mutations in seven of the 29, however, modify that of the alkanes (von Wettstein-Knowles, unpublished). The latter is expected given that in barley at least 19 genes with homology to FAE KCS6 occur (Weidenbach *et al.*, 2014), and that at least three sequential FAE-like complexes are required to synthesize C_32_ acyl chains from C_18_ precursors in barley spikes (Mikkelsen, 1978). The organization of all the mentioned elongation complexes, or metabolons (Laursen *et al.*, 2015), and how the growing acyl chain passes from one to the next is a fascinating question to be deciphered in the future.

**Fig. 6.**
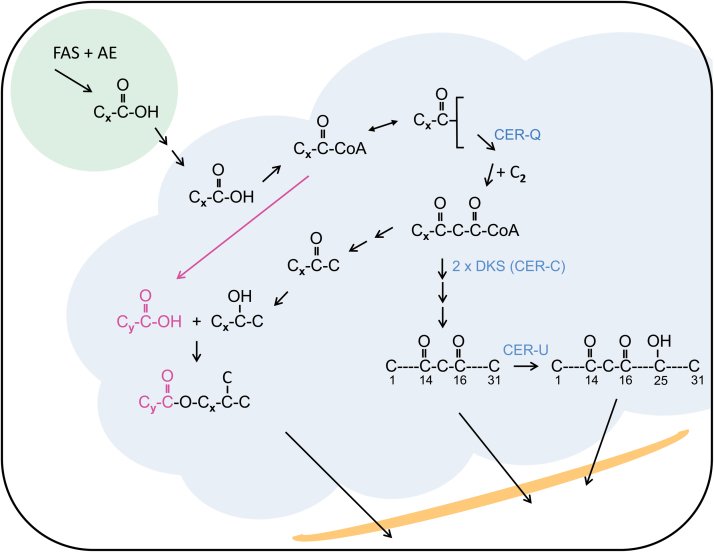
Proposed β-diketone synthase (DKS) polyketide pathway for synthesis of β-diketones, their derivatives and esterified alkan-2-ols. Fatty acid synthase (FAS) plus auxiliary enzymes (AE) in plastids (green) synthesize acyl chains (*x*=9–17) that are exported therefrom and presumed to enter the endoplasmic reticulum’s membrane (gray) where FAE derived aliphatics are synthesized. Here they are activated by CoA to form an acyl-CoA pool. Acyl editing transfers the acyl chains to a glycerolipid in a reversible reaction. Acyl-CoAs can serve as substrates for fatty acid elongase (FAE) complexes giving, for example, a fatty acid (*y*=17–21; pink, center left side). Given that CER-Q on the basis of homology is classified as a lipase, its acyl substrate is potentially esterified to a glycerolipid (top center). The CER-Q cleaved acyl chain activated by coenzyme A is elongated (+C_2_) to give the β-ketoacyl compound that is the substrate for two pathways; (i) β-diketones and their derivatives (right side). The DKS (CER-C) introduces two oxygens into the acyl chain, which are followed by further elongations analogous to those carried out by FAE and loss of a carbon yields β-diketones (see Fig. 1). In barley CER-U a P450 hydroxylase inserts a hydroxyl group on carbon 25. (ii) Esterified alkan-2-ols (left side). Cleavage of CoA plus the carboxyl carbon yields methyl ketones that can be hydrolyzed to short alkan-2-ols, primarily with 13 and 15 carbons, for esterification with fatty acids originating from FAE. Mutants of *Cer-u* and *Cer-c* accumulate β-diketones and esterified alk-2-ols, respectively. The final aliphatics are transported to and through the apoplast (orange) onto its surface. Single arrows, known reaction(s); sequential arrows, hypothetical reaction(s).

The present identification of the members of the *Cer-cqu* gene cluster has established that a polyketide pathway synthesizing β-diketone aliphatics can make significant contributions to the epicuticular wax layer on given plant apoplasts. These three genes affect only the β-diketone polyketide pathway aliphatics (Fig. 6). Initial elongation steps carried out by FAS take place in plastids, and the resulting acyl chains (10–18) are presumably exported to the ER, the site of FAE and the associated enzymes producing FAE derived aliphatics (Lee and Suh, 2015). An FAE-KCS conceivably gives the β-ketoacyl-CoA substrate for the DKS, and FAE components may also be required for the six final elongations to give the β-diketone carbon skeletons (Fig. 1). Moreover, that some of the acyl chains from FAE (Fig. 6, pink) are esterified to alkan-2-ols suggests that the synthetic machinery for the latter as well as for the β-diketone aliphatics is also present in the ER. Finally, all the elements necessary for initiating transport of the polyketide pathway aliphatics to the cuticle surface are present in the ER. These indirect clues that the *Cer-c*, *-q* and *-u* encoded proteins are associated with the ER awaits confirmation.

The proposed pathway in Fig. 6 also intimates that acyl chains destined for the β-diketone polyketide pathway are potentially sequestered from the general acyl-CoA pool used by FAE by being esterified to a glycerolipid. Transfer of acyl chains to TAG is well established in plants. CER-Q, classified as a lipase on basis of homology, would cleave them substrate specifically. Which glycerolipid and the time at which the elongation giving the β-ketoaycl-CoA DKS substrate takes place are at present unknown and will prove an intriguing research objective in the future. The proposed cleavage of CoA from the same β-ketoacyl-CoA substrate followed by a decarboxylation as takes place in synthesis of methylketones in tomatoes (Yu *et al.*, 2010) is in accord with early biosynthetic studies, as is the ensuing reduction to alkan-2-ols and their esterification (Mikkelsen, 1984). Cytochrome P450 enzymes are members of all plant gene clusters described to date, as they are tailoring enzymes giving rise to a diverse array of specialized metabolites (Boycheva *et al.*, 2014). CER-U hydroxylates carbon 25 of the C_31_ β-diketone chain, as do most of its homologs in the β-diketone polyketide pathway in Triticeae species. In some few species, however, alcohol groups occur on carbons-26, −4, −5+6 or −8+9 (Tulloch *et al.*, 1980) illustrating the capacity of the P450s to give rise to new metabolites.

### Phylogenetic aspects of the *Cer-cqu* gene cluster

The phylogenetic analysis of the CER-C, -Q and -U proteins showed that the most similar proteins belong to members of the Triticeae tribe, while proteins from other monocots and dicots showed a higher similarity to other barley proteins. This suggests a recent functional diversification of the *Cer-c*, *-q* and *-u* genes within the Triticeae after *Brachypodium* diverged from the last common ancestor ~32–39 million years ago (Middleton *et al.*, 2014). Since the same β-diketone aliphatics and esterified alkan-2-ols have also been documented in distant dicot relatives such as *Eucalyptus*, this would suggest that the ability to produce these compounds has evolved at least twice independently. This is true for the cyanogenic glucoside gene clusters in three different plants (Takos *et al.*, 2011). Alternatively, the *Cer-c*, *-q* and *-u* genes could be ancestral and present in all monocots and were subsequently lost in many species, e.g. rice, maize and *Brachypodium*, all of which lack β-diketone aliphatics and esterified alkan-2-ols. Perhaps the most interesting gene is *Cer-q*, which homology assigns to a relatively small set of enzymes whose functions remain relatively unexplored. This is in marked contrast to the extraordinary diversity of type III chalcone synthases and P450s whose diversity has been documented to play significant roles in a large number of different secondary metabolic pathways. The only potentially significant homologs of CER-Q were found in foxtail millet and sorghum. The leaf epicuticular wax of the latter lacks β-diketone aliphatics, but produces esterified alkan-2-ols implying a loss of the *Cer-c* homolog in this species. Is the same true for foxtail millet? Given that esterified alkan-2-ols do not make any contribution or at best make only a minor contribution to the plant phenotype, as judged from the *cer-c* mutants where they are major components, a more detailed chemical phenotypic analysis across species is necessary to elucidate their evolutionary relationships. An analogous study might also resolve the question of loss of functions from a single evolutionary event versus independent ones.

In wheat, production of β-diketones is genetically defined by three loci (*W1*, *W2* and *W3*) mapped to chromosome arms 2BS and 2DS (Tsunewaki and Ebana, 1999; Lu *et al.*, 2015; Zhang *et al.*, 2015). The closest wheat orthologs of *Cer-c*, *-q* and *-u* are all defined by gene models which have been assigned to these regions (IWGSC, 2014), which are syntenic to barley 2HS. This raises the possibility that *W1*, *W2* and/or *W3* might be homologs of the *Cer-c* or *-q* members of the barley gene cluster. This is further supported by molecular genetic evidence since the *Cer-cqu* members (this study) and the wheat *W1* locus have been mapped to an equivalent syntenic interval based on common markers (Adamski *et al.*, 2013; unpublished data). Whether *W1* and *W2* in wheat correspond to single genes or gene clusters such as *Cer-cqu* in barley can be tested now that the latter genes have been identified.

### The *Cer-cqu* mutant collection

In the present study the knowledge of the predicted function of two of the three genes plus the availability of large deletion mutants were critical for success as the *Cer-cqu* cluster lies in one of the many regions that have yet to be properly assembled despite the draft genome sequence (IBSC, 2012). To confirm that the correct gene has been identified, however, mutant collections are very valuable, and in barley 80 genes with a minimum of six mutants are available (Søgaard and von Wettstein-Knowles, 1987). For the validation of the *Cer-c*, -*q* and -*u* candidate genes a set of mutants (57+57+54, respectively), created using a range of different mutagens, was used which can be grouped into the two categories: (i) chemical mutagens and (ii) ionizing radiation. This strategy should produce a diverse allelic series given that chemical mutagens preferentially cause base alterations (e.g. G→A) while radiation mostly causes base deletions resulting in point mutations (DNA repair error), frameshift mutations (small deletion or addition in coding sequence) or deletions of large chromosomal segments. The 159 mutations identified in the three genes are consistent with these expectations. In 97.5% (115) of lines treated with chemical mutagens a point mutation was observed, while only two frameshift mutations and a single deletion could be detected. Similarly, in the mutant lines treated with ionizing radiation the observed mutations were more diverse, with 44% (11) being point mutations, 36% (9) frameshift mutations and 20% (5) large deletions. Interestingly, the mutants are essentially randomly distributed with the same mutation only occurring twice 15 times and treble two times out of 159, and no hot spots (Rogozin and Pavlov, 2003) materialize as did in Benzer’s 1961 analysis in which only two types of chemical mutagens were used (Benzer, 1961) neither of which was used to generate the *cer* mutant collection.

## Supplementary data

Supplementary data are available at *JXB* online.

Table S1. Detailed information on *eceriferum* mutants used in this study.

Table S2. Primers used in this study.

Fig. S1. Phylogenetic analysis of CER-C related proteins.

Fig. S2. Phylogenetic analysis of CER-Q related proteins.

Fig. S3. Phylogenetic analysis of CER-U related proteins.

Data S1. Comparative homology model of CER-C.

Data S2. Comparative homology model of CER-Q.

Data S3. Comparative homology model of CER-U.

Data S4. Pymol session showing CER-C with mutants labeled according to substitution and source mutant strain.

Data S5. Pymol session showing CER-Q with mutants labeled according to substitution and source mutant strain.

Data S6. Pymol session showing CER-U with mutants labeled according to substitution and source mutant strain.

Supplementary Data
