# The *Cer-cqu* gene cluster determines three key players in a β-diketone synthase polyketide pathway synthesizing aliphatics in epicuticular waxes

**DOI:** 10.1093/jxb/erw229

**Published:** 2016-06-02

**Authors:** Lizette M Schneider, Nikolai M Adamski, Caspar Elo Christensen, David B Stuart, Sonia Vautrin, Mats Hansson, Cristobal Uauy, Penny von Wettstein-Knowles

**Affiliations:** 1 Biology Department, Copenhagen University, Copenhagen, Denmark; 2 Biology Department, Lund University,Lund, Sweden; 3 John Innes Centre, Norwich Research Park, Norwich, UK; 4 INRA–Centre National de Ressources Génomiques Végétales, Castanet Tolosan, France

*Journal of Experimental Botany*, Vol. 67, No. 9, pp. 2715–2730, 2016, doi:10.1093/jxb/erw105

The authors of the above paper would like to state that Lizette M Schneider and Nikolai M Adamski contributed equally to this work. The online version has been corrected to reflect this.

