# Supplementary material for: The Cer-cqu gene cluster determines three key players in a β-diketone synthase polyketide pathway synthesizing aliphatics in epicuticular waxes
Source: J Exp Bot. 2016 Mar 9;67(9):2715–30. doi: 10.1093/jxb/erw105 (PMC4861019; doi:10.1093/jxb/erw105)
Supplement: Supplementary Data [file supp_67_9_2715__index.html]

The Cer-cqu gene cluster determines three key players in a β-diketone synthase polyketide pathway synthesizing aliphatics in epicuticular waxes — Supplementary Data 

# The *Cer-cqu* gene cluster determines three key players in a β-diketone synthase polyketide pathway synthesizing aliphatics in epicuticular waxes

## Supplementary Data

Data files

- supplementary\_figures\_S1\_S3.pdf - Supplementary Data
- supplementary\_table\_S1.xlsx - Supplementary Data
- supplementary\_table\_S2.xlsx - Supplementary Data
- Supp\_Data\_S1.pdb - Supplementary Data
- Supp\_Data\_S2.pdb - Supplementary Data
- Supp\_Data\_S3.pdb - Supplementary Data
- Supp\_Data\_S4.pse - Supplementary Data
- Supp\_Data\_S5.pse - Supplementary Data
- Supp\_Data\_S6.pse - Supplementary Data
