# Supplementary material for: The Cer-cqu gene cluster determines three key players in a β-diketone synthase polyketide pathway synthesizing aliphatics in epicuticular waxes
Source: J Exp Bot. 2016 Mar 9;67(9):2715–30. doi: 10.1093/jxb/erw105 (PMC4861019; doi:10.1093/jxb/erw105)

SUPPLEMENTARY DATA

Materials and Methods

PSSM and SIFT analysis:
The PARSESNP (http://blocks.fhcrc.org/proweb/input/) and Blockmaker (Henikoff et al. 2000) programs were used to generate a set of blocks/motifs, which were used for effect prediction of nucleotide changes using the Position-Specific Scoring Matrix (PSSM) and Sorting Intolerant From Tolerant (SIFT) scores. Only nucleotide changes that occured within these blocks were assessed by the PSSM algorithm (81 of the 106 non-synoymous mutations). PSSM differences above 10 and SIFT scores below 0.05 are predicted to be deleterious.

Figures

Supplementary Figure S1:
Phylogenetic analysis of CER-C related proteins across monocot species available at Phytozome and EnsemblPlants. The *Triticeae*-specific clade including CER-C (MLOC_59804.1) is shown in blue; while the monocot clade excluding rice is in green and the monocot clade including rice is in yellow. For detailed description of species used, alignment and tree construction methods see Materials and Methods.

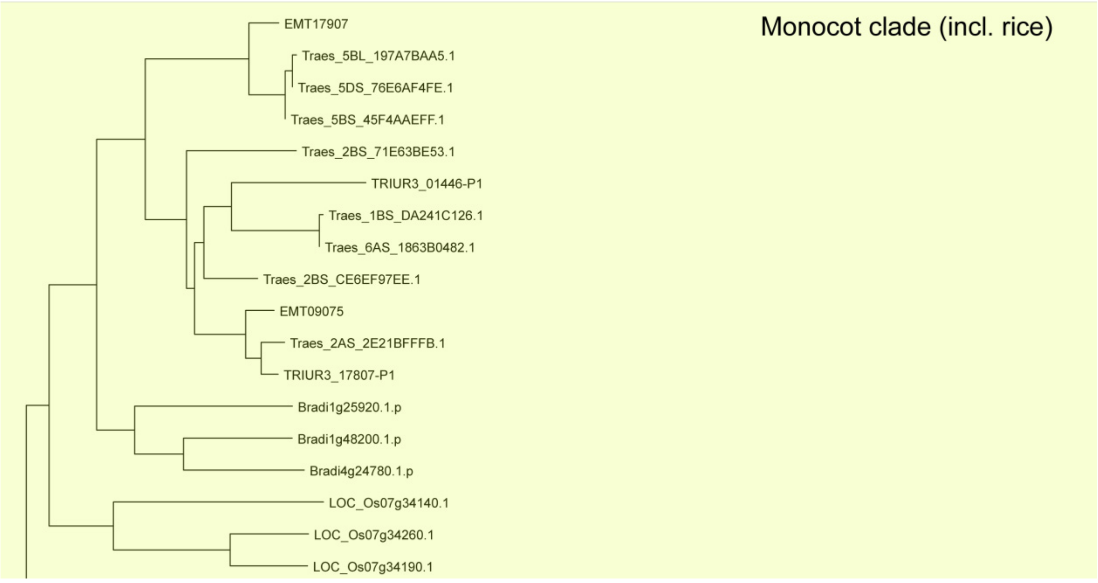

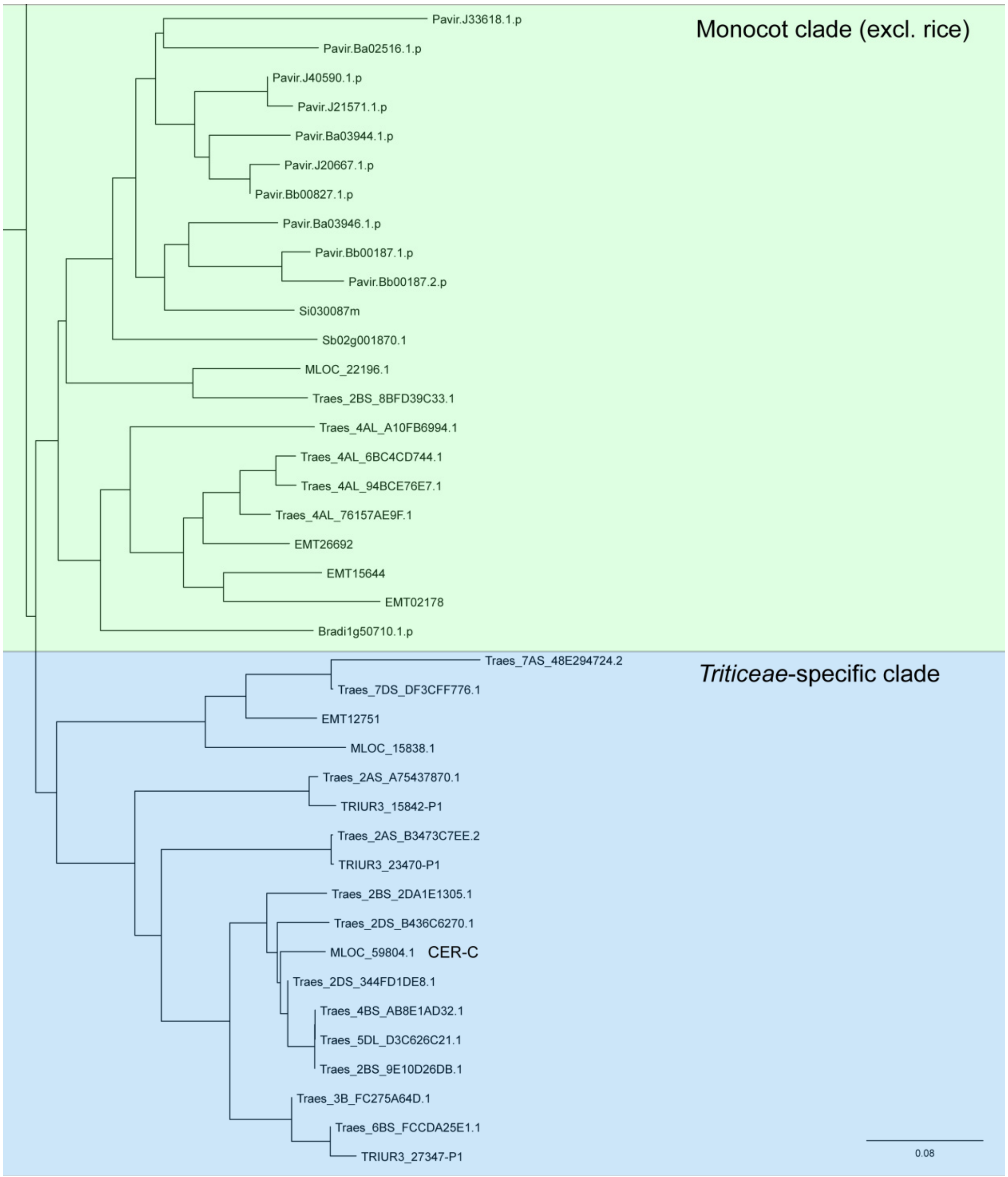
Monocot clade (excl. rice)
Pavir.J33618.1.p
Pavir.Ba02516.1.p
Pavir.J40590.1.p
Pavir.J21571.1.p
Pavir.Ba03944.1.p
Pavir.J20667.1.p
Pavir.Bb00827.1.p
Pavir.Ba03946.1.p
Pavir.Bb00187.1.p
Pavir.Bb00187.2.p
Si030087m
Sb02g001870.1
MLOC_22196.1
Traes_2BS_8BFD39C33.1
Traes_4AL_A10FB6994.1
Traes_4AL_6BC4CD744.1
Traes_4AL_94BCE76E7.1
Traes_4AL_76157AE9F.1
EMT26692
EMT15644
EMT02178
Bradi1g50710.1.p
Triticeae-specific clade
Traes_7AS_48E294724.2
Traes_7DS_DF3CFF776.1
EMT12751
MLOC_15838.1
Traes_2AS_A75437870.1
TRIUR3_15842-P1
Traes_2AS_B3473C7EE.2
TRIUR3_23470-P1
Traes_2BS_2DA1E1305.1
Traes_2DS_B436C6270.1
MLOC_59804.1
CER-C
Traes_2DS_344FD1DE8.1
Traes_4BS_AB8E1AD32.1
Traes_5DL_D3C626C21.1
Traes_2BS_9E10D26DB.1
Traes_3B_FC275A64D.1
Traes_6BS_FCCDA25E1.1
TRIUR3_27347-P1
0.08

Supplementary Figure S2:
Phylogenetic analysis of CER-Q related proteins across monocot species available at Phytozome and EnsemblPlants. The *Triticeae*-specific clade including CER-Q (MLOC_13397.1) is shown in blue; the monocot clades excluding rice are shown in green and pink; and the monocot clade including rice is in yellow. For detailed description of species used, alignment and tree construction methods see Materials and Methods.

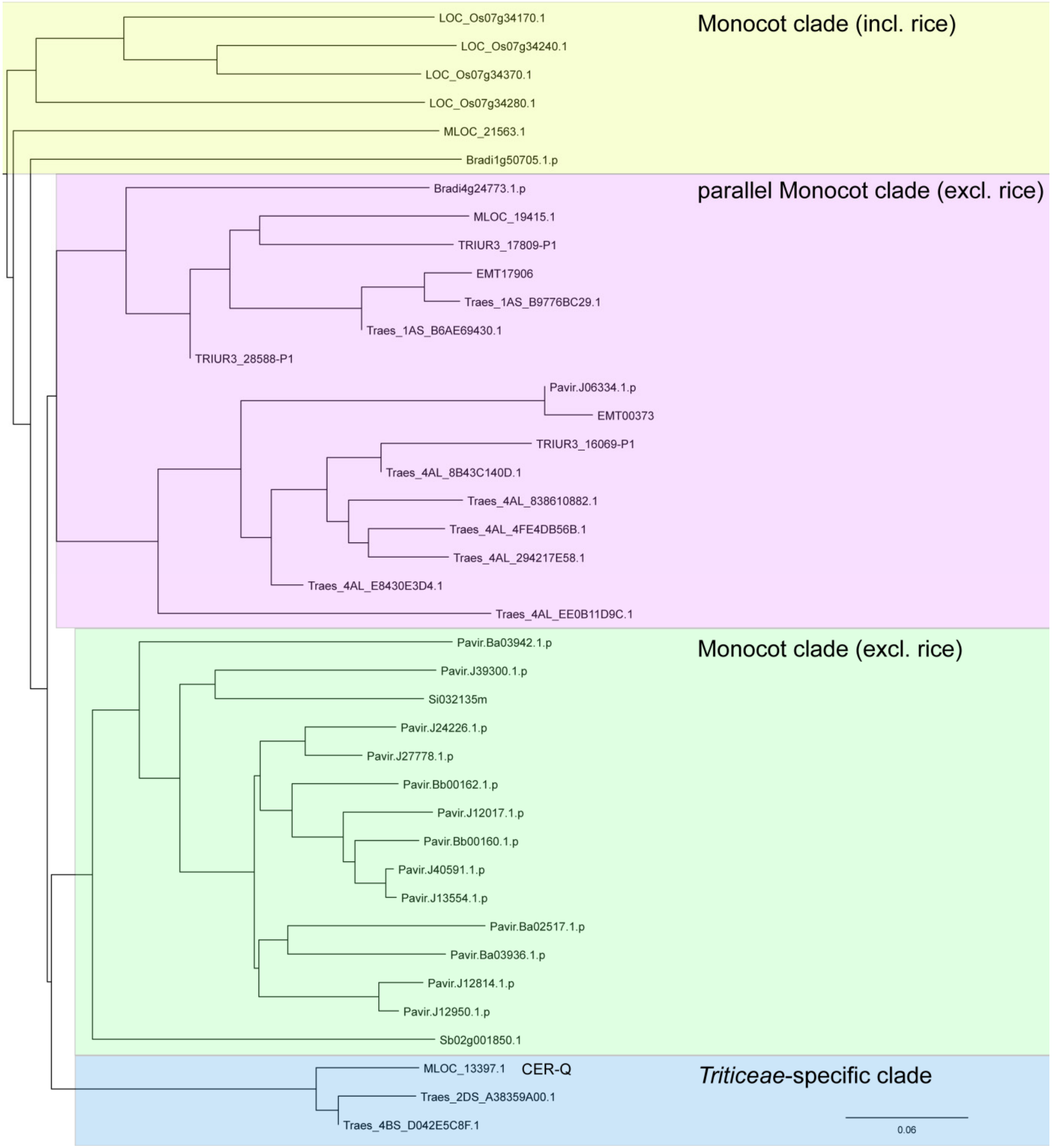

Supplementary Figure S3:
Phylogenetic analysis of CER-U related proteins across monocot species available at Phytozome and EnsemblPlants. The *Triticeae*-specific clade including CER-U (AK373499) is shown in blue; while the monocot clade excluding rice is in green and the monocot clade including rice is in yellow. For detailed description of species used, alignment and tree construction methods see Materials and Methods.

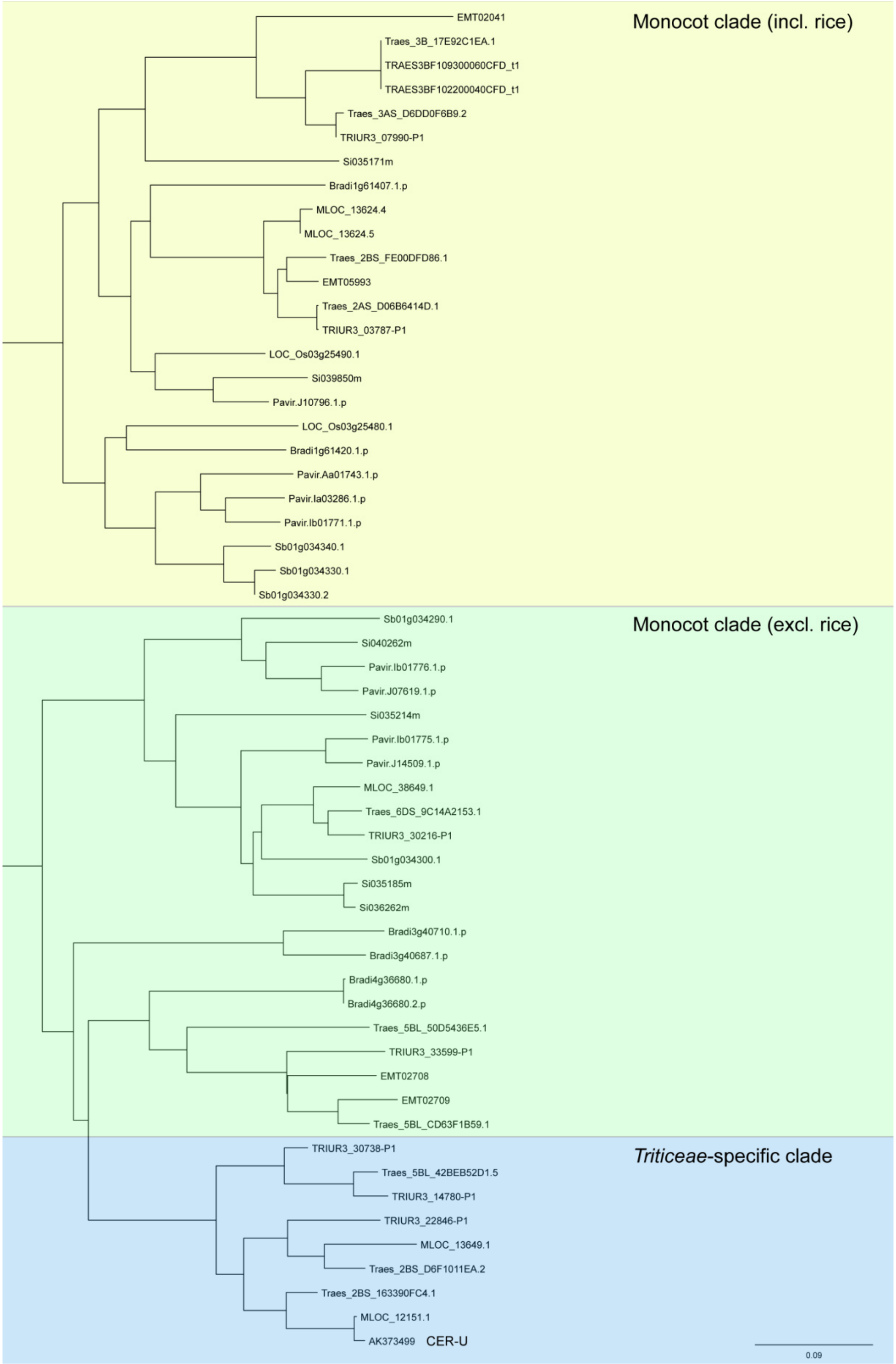

Supplement: Supplementary Data [file supp_erw105_supplementary_figures_S1_S3.pdf]
